# Impact of Extremely Low-Frequency Electromagnetic Fields on Skeletal Muscle of Sedentary Adult Mice: A Pilot Study

**DOI:** 10.3390/ijms25189857

**Published:** 2024-09-12

**Authors:** Caterina Morabito, Noemi Di Sinno, Maria A. Mariggiò, Simone Guarnieri

**Affiliations:** 1 Department of Neuroscience, Imaging and Clinical Sciences, University “G. d’Annunzio” of Chieti-Pescara, 66100 Chieti, Italy; caterina.morabito@unich.it (C.M.); disinnon@gmail.com (N.D.S.); simone.guarnieri@unich.it (S.G.); 2 Center for Advanced Studies and Technology (CAST), University “G. d’Annunzio” of Chieti-Pescara, 66100 Chieti, Italy

**Keywords:** ELF-EMF, muscle regeneration, antioxidant, oxidative stress

## Abstract

Extremely low-frequency electromagnetic fields (ELF-EMFs) are ubiquitous in industrialized environments due to the continuous use of electrical devices. Our previous studies demonstrated that ELF-EMFs affect muscle cells by modulating oxidative stress and enhancing myogenesis. This pilot study investigated these effects on the skeletal muscles of sedentary adult mice, assessing physiological responses to ELF-EMF exposure and potential modulation by antioxidant supplementation. Male C57BL/6 mice were exposed to ELF-EMFs (0.1 or 1.0 mT) for 1 h/day for up to 5 weeks and fed a standard diet without or with N-acetyl-cysteine (NAC). The results showed transient increases in muscle strength (after 2 weeks of exposure at 1.0 mT), potentially linked to muscle fiber recruitment and activation, revealed by higher PAX7 and myosin heavy chain (MyH) expression levels. After ELF-EMF exposure, oxidative status assessment revealed transient increases in the expression levels of SOD1 and catalase enzymes, in total antioxidant capacity, and in protein carbonyl levels, markers of oxidative damage. These effects were partially reduced by NAC. In conclusion, ELF-EMF exposure affects skeletal muscle physiology and NAC supplementation partially mitigates these effects, highlighting the complex interactions between ELF-EMFs and antioxidant pathways in vivo. Further investigations on ELF-EMFs as a therapeutic modality for muscle health are necessary.

## 1. Introduction

Humans are constantly exposed to electromagnetic fields originating from both natural and artificial sources. Extremely low-frequency electromagnetic fields (ELF-EMFs), ranging from 3 Hz to 300 Hz and produced by power lines, railways, and electrical devices, have been the subject of extensive biomedical research. However, the information available remains fragmentary, incomplete, and occasionally contradictory, leaving uncertainty about whether exposure to ELF-EMFs is beneficial or harmful to the human body [1,2,3,4].

Numerous studies have suggested potential genotoxic, carcinogenic, and neurological effects induced by ELF-EMFs [5,6,7], but recent investigations have also reported anti-neoplastic effects [8,9,10]. A recent study highlighted that ELF-EMF exposure can inhibit cell growth and enhanced both coupled and uncoupled respiration, with significant metabolic shifts in the 3D cell cultures of human pancreatic cancer, glioblastoma, and breast cancer [11]. In addition, Moori et al. demonstrated, on breast cancer cells, that ELF-EMF exposure significantly reduced cell migration and invasion by modulating cadherin switching, increasing E-cadherin, and decreasing N-cadherin expression. This suggests that ELF-EMFs can suppress metastasis through the regulation of epithelial–mesenchymal transition pathways [12]. The beneficial effects of ELF-EMFs have also been investigated in various organs and body systems, including the mechanisms of repair in bone, cartilage, skin, and skeletal muscle [13,14,15,16]. Also, ELF-EMF therapy showed promising benefits in enhancing functional recovery after stroke, with efficacy dependent on specific frequencies. In a mouse cortical stroke model, a 5 Hz ELF-EMF significantly improved motor recovery, while higher frequencies showed no effect [17].

This heterogeneity of results could be primarily attributed to variable models and different frequencies, intensities, and exposure times of ELF-EMFs.

To fully comprehend the effects of electromagnetic fields on skeletal muscle and other body systems, both in vitro and in vivo models are required. In vitro models provide a controlled environment (including specific wavelengths, frequencies, and magnetic field intensities) to examine the direct effects of ELF-EMFs on cells, allowing a focused understanding of the cellular mechanisms involved.

Our previous in vitro investigations provided new insights into the mechanisms underlying the biological effects of ELF-EMFs on skeletal muscle. We demonstrated that short exposures (5–30 min) to ELF-EMFs (0.1–1.0 mT, 50 Hz) on skeletal muscle cells modulate their redox status and Ca^2+^ handling and that these effects are abolished by the administration of an antioxidant molecule [18]. Many other in vitro studies revealed that the mechanisms underlying the interaction between ELF-EMFs and cells involve the production of reactive oxygen species (ROS) [19,20,21]. If ROS generation and accumulation in the cells can be considered the primum movens of effects induced by ELF-EMF exposure, the modification of intracellular Ca^2+^ levels could be one of the most important mechanisms by which ROS exert their multiple effects on cellular processes, including proliferation, apoptosis, and differentiation [22,23,24,25]. Furthermore, a recent study demonstrated that the ELF-EMF exposure (1 mT for 24 and 48 h) of adipose-derived stem cells induced significant epitranscriptomic changes, notably increasing RNA methylation. This also enhanced the expression of stem cell markers and altered cellular metabolism, mitochondrial morphology, and membrane flexibility, conditioning cell fate [26].

Indeed, we observed that long exposures (24-48-72 h) to ELF-EMFs promoted skeletal muscle differentiation in C2C12 cells through increasing gap junctional intercellular communication and facilitating the direct cell-cell transfer of ions such as Ca^2+^ and other small molecules [27].

A recent study found that exposure to ELF-EMFs at a 2.0 mT magnetic flux density can activate C2C12 cells and upregulate the transcription factor PAX7, which is crucial for the activation and proliferation of satellite cells. Meanwhile, exposure to a 1.5 mT ELF-EMF can upregulate the expression of MyoD and myogenin. The authors suggest that ELF-EMF therapy that uses different magnetic flux densities may be more effective in promoting muscle repair in clinical practice [28].

Nevertheless, in vitro models have limitations, as they simplify the natural environment and overlook the complex interactions between various cell types, tissues, and organs found in living organisms. Animal models can provide a more integrated understanding of the physiological consequences of electromagnetic field exposure on skeletal muscle since they allow the observation of long-term effects and the exploration of complex interactions between different organs and tissues.

Some in vivo studies have confirmed that the action of ELF-EMFs occurs through the production of ROS [3,29]. 

Lai et al. reported that the exposure of rats to a 60 Hz magnetic field induced DNA single- and double-bond breaks in nervous cells [30,31]. The use of free radical scavengers prevented DNA damage, thus supporting the hypothesis of an active role of free radicals in EMF-induced processes. In a similar study conducted by Gao and colleagues, natural antioxidants, catechin and epicatechin, showed an ability to protect animals’ brains from oxidative stress induced by ELF-EMFs at 50 Hz [32].

Moreover, Gunes and collaborators [33] observed that the muscle mechanical activity of the diaphragm was not affected in rats exposed to sinusoidal ELF-EMFs (50 Hz frequency, 1.5 mT magnetic flux density) from the neonatal to adult periods (chronic exposure).

While studies have been conducted on the effects of ELF-EMFs on various tissues and functions, there is still limited research on the potential consequences of ELF-EMFs on skeletal muscle function. Considering our previous in vitro results and those from other authors, the aim of this work is to study ELF-EMFs’ impact on skeletal muscle. The experimental plane was designed using C57BL/6 sedentary adult mice exposed to 0.1 or 1 mT ELF-EMFs (50 Hz) for 1 h/day for up to 5 weeks. During the experimental period, control (sham group) and exposed animals were fed a standard diet or an N-acetyl-cysteine (NAC)-enriched diet. 

Understanding the mechanisms underlying the effects induced by ELF-EMFs on skeletal muscle can provide valuable insights into the potential therapeutic applications of electromagnetic fields.

## 2. Results

### 2.1. Effects of ELF-EMF Exposure on Body Weight and Muscle Strength

**Figure 1:**
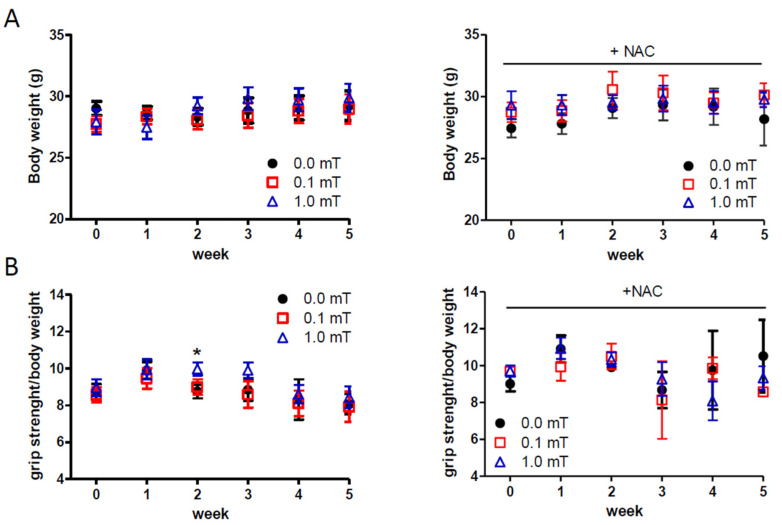
In vivo measurements. (**A**) Body weight and (**B**) muscle strength measurements in mice that experienced 0.0, 0.1, or 1.0 mT ELF-EMFs for up to 5 weeks, during which the mice were fed a standard diet (**left panels**) or a diet supplemented with 1% NAC in drinking water (**right panels**). Body weights (g) and muscle strengths (expressed as ratio between grip strength and body weight of each mouse) were measured weekly. All data are expressed as means ± S.E.M. from six mice/group. * *p* < 0.05 versus 0.0 mT without NAC supplementation.

During the experimentation (see the figure in Section 4.2 and Section 4), the body weight and skeletal muscle strength of the mice were monitored in all conditions in order to test in vivo macroscopic features related to skeletal muscle health. No significant difference in body weights was observed among different experimental groups at each time point (Figure 1A). Muscle strength data showed a transient significant increase of muscle strength in the group of mice exposed to a 1.0 mT ELF-EMF and fed a standard diet compared to the sham group (0.0 mT) fed a standard diet as well (Figure 1B). No other differences in muscle strength were observed in groups fed a standard diet. Muscle strength did not show significant differences between the sham group (0.0 mT) and ELF-EMF-exposed groups (0.1, and 1.0 mT) fed a diet with N-acetyl-cysteine (NAC) supplementation (Figure 1B, left panel). These results suggested that exposure to 0.1 and 1.0 mT ELF-EMFs did not significantly affect body weight, and consequently muscle mass, in all experimental groups. Although muscle strength remained largely unaffected, a transitory increase was observed in the group exposed to a 1.0 mT ELF-EMF after two weeks, hypothesizing an impact, albeit limited, of ELF-EMF on muscle function. 

### 2.2. ELF-EMF Exposures Modulate Skeletal Muscle Regeneration Markers

Considered the above-mentioned results of in vivo measurements, we were incouraged to investigate the influence of ELF-EMFs on the molecular markers of muscle trophism and regeneration in order to check signs of skeletal muscle responsiveness/adaptation to ELF-EMF daily exposure.

**Figure 2:**
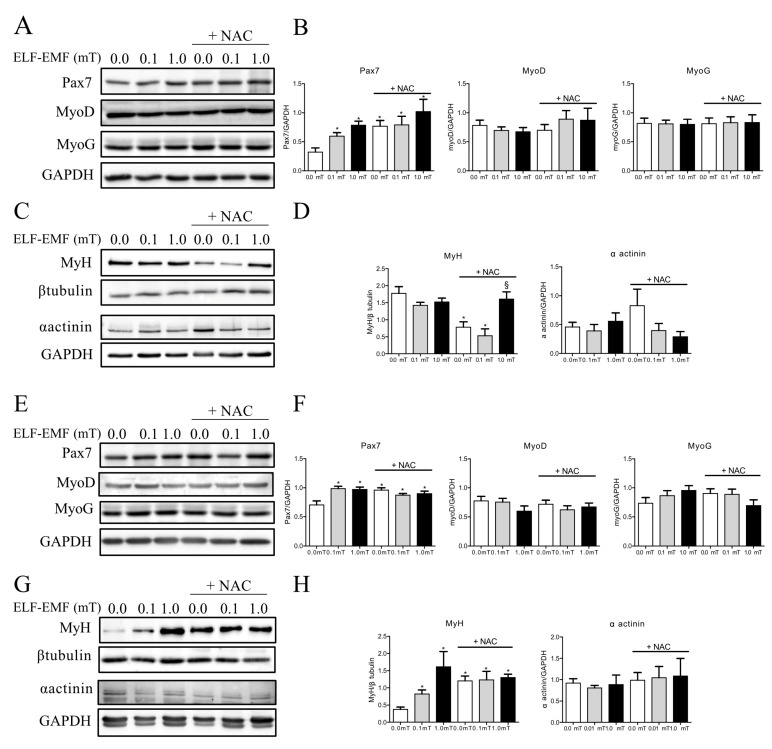
Expression levels of muscle regeneration markers. Proteins’ expression levels were assayed by Western blot analyses in skeletal muscles from mice groups. (**A**) Representative immunoblots of Pax7, MyoD, and myogenin (MyoG), in skeletal muscles from mice groups after 1-week exposure (0.0, 0.1, or 1.0 mT ELF-EMF) and that were fed a diet without or with (+NAC) NAC supplementation. (**B**) The corresponding densitometric analyses of Western blot represented in panel (**A**). (**C**) Representative immunoblots of MyH and α-actinin, in skeletal muscles from mice groups after 1-week exposure (0.0, 0.1, or 1.0 mT ELF-EMFs) and that were fed a diet without or with (+NAC) NAC supplementation. (**D**) the corresponding densitometric analyses of Western blot represented in panel (**C**). (**E**) Representative immunoblots of Pax7, MyoD, and myogenin (MyoG), in skeletal muscles from mice groups after 5 weeks of exposure (0.0, 0.1, or 1.0 mT ELF-EMFs) and that were fed a diet without or with (+NAC) NAC supplementation. (**F**) the corresponding densitometric analyses of Western blot represented in panel (**E**). (**G**) Representative immunoblots of MyH and α-actinin, in skeletal muscles from mice groups after 5 weeks of exposure (0.0, 0.1, or 1.0 mT ELF-EMFs) and that were fed a diet without or with (+NAC) NAC supplementation. (**H**) the corresponding densitometric analyses of Western blot represented in panel G. All densitometry analyses (**B**,**D**,**F**,**H**) were calculated as the ratio between the OD × mm^2^ of each band and the OD × mm^2^ of the corresponding loading control band (GAPDH for Pax7, MyoD, MyoG, and α-actinin, or β-tubulin for MyH). All data are expressed as means ± S.E.M. from six independent experiments. * *p* < 0.05 versus 0.0 mT without NAC supplementation. § *p* < 0.05 versus 0.0 mT with NAC supplementation.

The effects of ELF-EMF treatments on muscle regeneration were evaluated by testing the expression levels of some myogenesis markers, such as Pax7, MyoD, myogenin (MyoG), myosin heavy chain (MyH), and α-actinin, which were considered early (PAX7 and MyoD), intermediate (MyoG), and late (MyH and α-actinin) muscle differentiation markers. As shown in Figure 2A,B, the expression levels of PAX7 showed significant slight increases in skeletal muscles from mice exposed to ELF-EMFs (0.1 mT or 1.0 mT) as well as in those from all groups supplemented with NAC compared to the sham group (0.0 mT) that was fed a diet without antioxidant supplementation. This increase was detectable not only after 1 week but also after 5 weeks of experimentation (Figure 2E,F). No changes in the expression levels of MyoD and MyoG were observed in any experimental conditions and exposure times (Figure 2A,B,E,F).

After 1 week, there was a significant reduction in MyH expression levels in skeletal muscles from mice of the sham group (0.0 mT) and from those exposed to a 0.1 mT ELF-EMF and both groups fed a diet with NAC supplementation, compared to samples from the sham group fed a standard diet without NAC (Figure 2C,D). In mice groups that were fed a diet with NAC supplementation, MyH expression levels increased in skeletal muscles from mice exposed to a 1.0 mT ELF-EMF compared to those from the corresponding sham group (0.0 mT) (Figure 2C,D). After 5 weeks, MyH expression levels showed a significant increase in skeletal muscles from mice exposed to ELF-EMFs (0.1 mT or 1.0 mT) and from all groups supplemented with NAC compared to the sham group that was fed a diet without NAC supplementation (Figure 2G,H). At the same experimental conditions and time points (1 and 5 weeks), the expression levels of α-actinin did not show any significant variations (Figure 2C,D,G,H).

The analysis of myogenesis markers revealed an increase in the early muscle regeneration marker Pax7 that started after 1 week of ELF-EMF exposure and was still detectable at 5 weeks. The early (1 week) and long-lasting (5 weeks) increases in Pax7 expression were followed, after 5 weeks, by the increased expression of the myogenesis late marker MyH, supporting the hypothesis that ELF-EMF exposure promotes the muscle regeneration process. In this scenario, antioxidant supplementation in the diet seemed to induce the same effect in skeletal muscle from the sham group, but it had no additive effects on muscle from ELF-EMF-exposed mice. These findings prompted us to explore how these exposure conditions could affect the oxidative status of skeletal muscles. 

### 2.3. ELF-EMF Exposure Modulates Oxidative Status in Mice Skeletal Muscle

**Figure 3:**
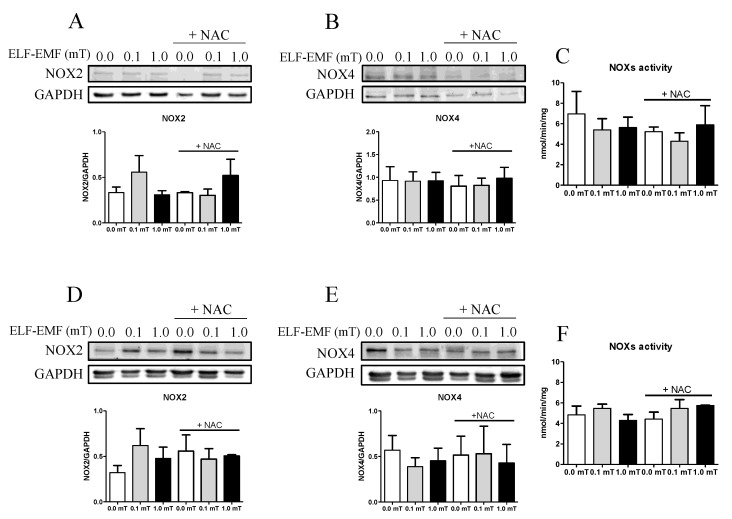
Pro-oxidant enzymes expression levels and activity. (**A**) Representative immunoblots of NOX2 and the corresponding densitometric analyses in skeletal muscle from mice groups after 1-week exposure (0.0, 0.1, or 1.0 mT ELF-EMFs) during which they were fed a diet without or with (+NAC) NAC supplementation. (**B**) Representative immunoblots of NOX4 and the corresponding densitometric analyses in the same skeletal muscle samples tested in (**A**). (**C**) NOX enzymatic activity (expressed as nmol NAPDH/min/mg protein) in the same skeletal muscle samples tested in (**A**,**B**). (**D**) Representative immunoblots of NOX2 and the corresponding densitometric analyses in skeletal muscle from mice groups after 5 weeks of exposure (0.0, 0.1, or 1.0 mT ELF-EMFs) during which they were fed a diet without or with (+NAC) NAC supplementation. (**E**) Representative immunoblots of NOX4 and the corresponding densitometric analyses in the same skeletal samples tested in (**D**). (**F**) NOX enzymatic activity (expressed as nmol NAPDH/min/mg protein) in the same skeletal samples tested in (**D**). All densitometric analyses (**A**,**B**,**D**,**E**) were calculated as the ratio between the OD × mm^2^ of each band and the OD × mm^2^ of the corresponding loading control band (GAPDH). All data are expressed as means ± S.E.M. from six independent experiments.

It is known that one of the targets of ELF-EMF exposure is the production of reactive oxygen species (ROS) in a skeletal muscle cell model [9]. The impact of ELF-EMF exposure on oxidative status in skeletal muscle was evaluated by investigating the expression and activity of ROS-generating enzymes as well as cellular ROS-detoxifying enzymes. Considering that NADPH oxidases (NOXs) are among the major producers of free radicals in skeletal muscle, their enzymatic activity was assayed, as well as the expression levels of NOX2 and NOX4 enzymes. After 1 and 5 weeks of experimentation (0.0, 0.1, and 1.0 mT ELF-EMF exposure) without or with (+NAC) NAC supplementation in the diet, the expression levels of NOX2 and NOX4 in skeletal muscle samples were not significantly different among all groups (Figure 3A,B,D,E). Similarly, NOX enzymatic activity assay, performed after 1 week or 5 weeks of experimentation, revealed no significant differences between the sham (0.0 mT) and the ELF-EMF (0.1 or 1.0 mT)-exposed groups, with or without NAC supplementation (Figure 3C,F). 

**Figure 4:**
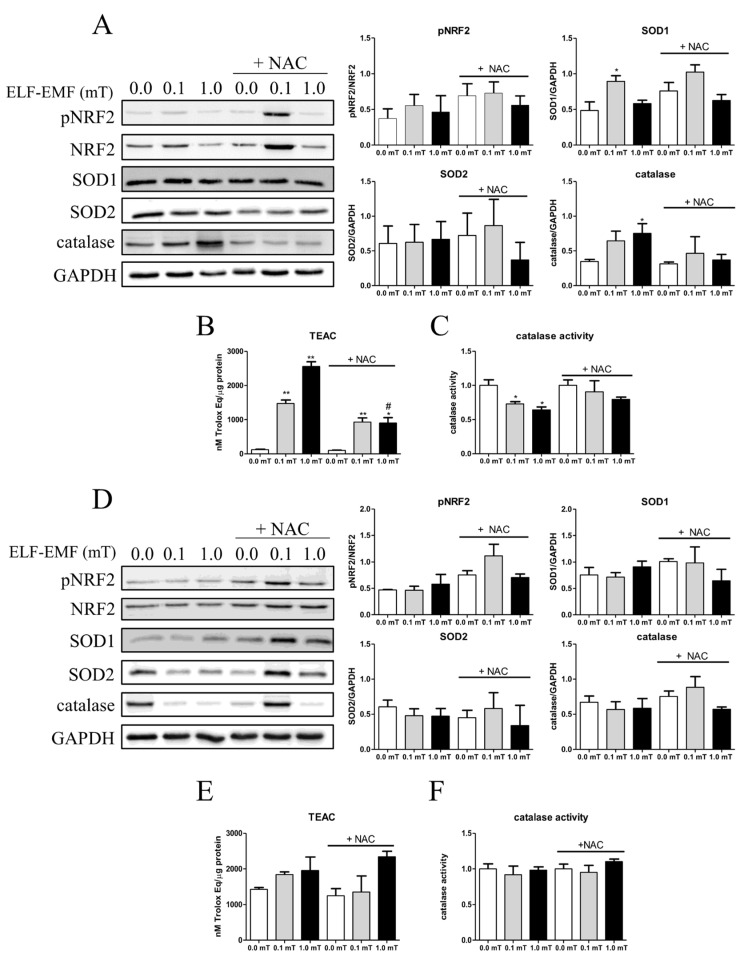
Anti-oxidative enzyme expression levels and activity. (**A**) Representative immunoblots of pNRF2, SOD1, SOD2, and catalase (**Left panels**) and the corresponding densitometric analyses (**Right panels**) in skeletal muscles from mice groups after 1-week exposure (0.0, 0.1, or 1.0 mT ELF-EMFs) during which they were fed a diet without or with (+NAC) NAC supplementation. (**B**) Total antioxidant status (expressed as nM Trolox equivalent/μg protein) measured by TEAC assay (see Section 4) in the same skeletal muscle samples tested in (**A**). (**C**) Catalase enzymatic activity (expressed as µmoles H_2_O_2_/min/mL) in the same skeletal muscle samples tested in (**A**). (**D**) Representative immunoblots of pNRF2, SOD1, SOD2, and catalase (**Left panels**) and the corresponding densitometric analyses (**Right panels**) in skeletal muscle from mice groups after 5 weeks of exposure (0.0, 0.1, or 1.0 mT ELF-EMFs), during which they were fed a diet without or with (+NAC) NAC supplementation. (**E**) Total antioxidant status (expressed as nM Trolox equivalent/μg protein) measured by TEAC assay (see Section 4) in the same skeletal muscle samples tested in (**D**). (**F**) Catalase enzymatic activity (expressed as µmoles H_2_O_2_/min/mL) in the same skeletal muscle samples tested in (**D**). All densitometric analyses were calculated as the ratio between OD × mm^2^ of each band and OD × mm^2^ of the corresponding loading control band (NRF2 for pNRF2, or GAPDH for SOD1, SOD2, and catalase). All data are expressed as means ± S.E.M. from six independent experiments. * *p* < 0.05 and ** *p* < 0.01 versus 0.0 mT without NAC supplementation; ^#^ *p* < 0.05 versus 0.0 mT with NAC supplementation.

In addition, the redox status of skeletal muscle was evaluated by analyzing the expression levels of key antioxidant enzymes (like SOD1, SOD2, catalase, and the transcription factor NRF2, which, when phosphorylated, is a regulator of cellular resistance to oxidants). After 1 week, the phosphorylated form of NRF2 and SOD2 expression levels did not change in skeletal muscle among all experimental groups (Figure 4A). On the other hand, the expression levels of SOD1, the enzyme converting cytoplasmic superoxide anions into hydrogen peroxide, and of catalase, which neutralizes hydrogen peroxide, were significantly increased in samples from mice that were fed a diet without NAC supplementation and exposed to 0.1 or 1.0 mT ELF-EMFs, respectively, when compared to samples from the sham group (Figure 4A). In addition, the catalase activity was significantly downregulated in samples from mice with a diet without NAC supplementation and exposed to 0.1 or 1.0 mT ELF-EMF in comparison to samples from the sham group (Figure 4C). The presence of NAC in the diet appeared to abolish these ELF-EMF-induced effects (Figure 4A,C). These findings, even if indirectly, suggested that 1 week of ELF-EMF exposure induced oxidative stress, and the presence of NAC in the diet had a potential antioxidant role against ELF-EMF. 

The total antioxidant capacity of samples, evaluated by TEAC assay, changed after ELF-EMF treatments. The results showed a significant increase in the total antioxidant capacity in samples from mice exposed to 0.1 or 1.0 mT ELF-EMFs compared to the sham group (0.0 mT). This effect was not affected by the presence or absence of NAC supplementation in the diet (Figure 4B). 

After the 5-week period of experimentation, no significant difference was observed in any tested features of the antioxidant machinery of skeletal muscles from mice kept in different conditions (Figure 4D–F). 

In skeletal muscle, the increased total antioxidant capacity after 1 week of ELF-EMF exposure and the unchanged oxidative status after 5 weeks of exposure are indicative of an early ELF-EMF-induced effect, followed by adaptation and recovery of the oxidative balance after 5 weeks. 

### 2.4. ELF-EMF Exposure Affects Oxidative Damage in Mice Skeletal Muscles

**Figure 5:**
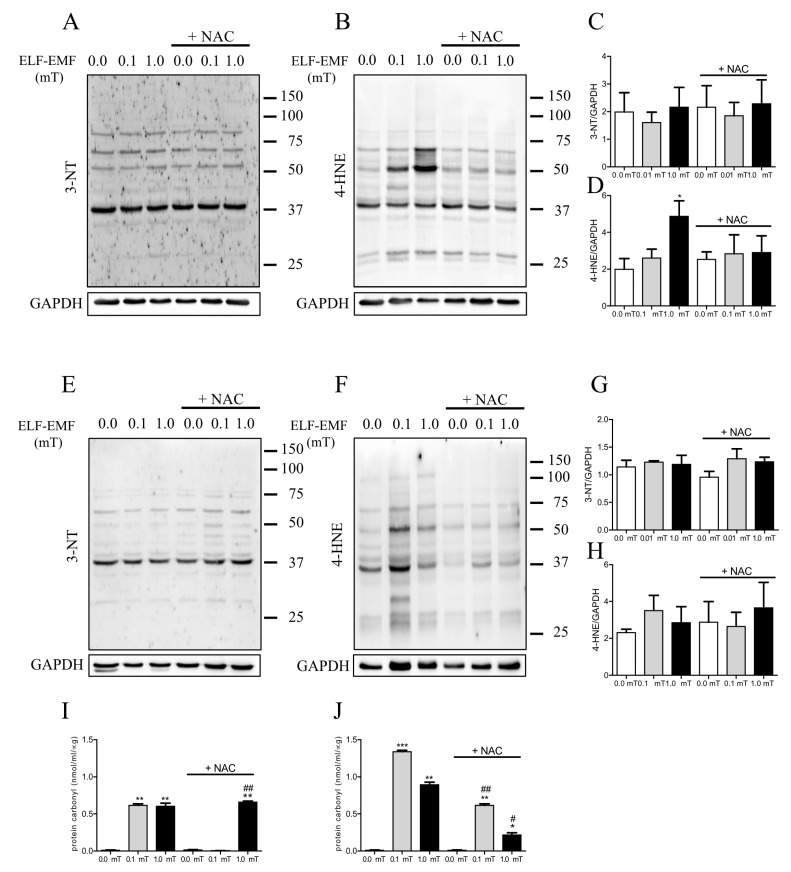
Expression of protein and lipid oxidative markers in skeletal muscles. (**A**) Representative immunoblots of the expression levels of 3-nitrotyrosine (3-NT) in skeletal muscle samples from mice groups after 1-week exposure (0.0, 0.1, or 1.0 mT ELF-EMFs) during which they were fed a diet without or with (+NAC) NAC supplementation. (**B**) Representative immunoblots of the expression levels of 4-hydroxynonenal (4-HNE) from skeletal muscle samples as described in (**A**). (**C**) Densitometric analyses of Western blots of samples shown in (**A**). (**D**) Densitometric analyses of Western blots of samples shown (**B**). (**E**) Representative immunoblots of the expression levels of 3-NT in skeletal muscle samples from mice groups after 5 weeks of exposure (0.0, 0.1, or 1.0 mT ELF-EMFs), during which they were fed a diet without or with (+NAC) NAC supplementation. (**F**) Representative immunoblots of the expression levels of 4-HNE in skeletal muscle samples as described in (**E**). (**G**) Densitometric analyses of Western blots of samples shown in (**E**). (**H**) Densitometric analyses of Western blots of samples shown in (**F**). (**I**) Protein carbonyl content in skeletal muscle samples as described in (**A**). (**I**,**J**) Protein carbonyl content in skeletal muscle samples as described in (**E**). The densitometric analyses were calculated as the ratio between the OD × mm^2^ of each band and the OD × mm^2^ of the corresponding GAPDH band, which was used as loading control. All data are expressed as means ± S.E.M. from six independent experiments. * *p* < 0.05, ** *p* < 0.01 and *** *p* < 0.001 versus 0.0 mT without NAC supplementation. ^#^ *p* < 0.05 and ^##^ *p* < 0.01 versus 0.0 mT with NAC supplementation.

Considering the impact of ELF-EMFs on the oxidative balance in skeletal muscle, we tested whether ELF-EMF exposure could induce oxidative damage in skeletal muscle. To this aim, three markers of oxidized macromolecules were assayed: 3-nitrotyrosine (3-NT) expression levels and protein carbonyl content as indicators of protein oxidation and 4-hydroxynonenal (4-HNE) as an indicator of lipid oxidation. Western analyses revealed that after 1 week of treatment, 4-HNE expression levels were significantly increased in skeletal muscle from mice exposed to a 1.0 mT ELF-EMF compared to the sham group. This effect was mitigated by NAC supplementation (Figure 5B,D) and was not present after 5 weeks of treatment (Figure 5F,H). No other significant differences were detected for 3-NT and 4-HNE expression levels in the other experimental conditions (Figure 5A–H). The content of protein carbonyls increased in skeletal muscle from mice exposed to ELF-EMFs (0.1 or 1.0 mT) both after 1 week and 5 weeks (Figure 5I,J). This effect was not present in skeletal muscles from mice exposed to a 0.1 mT ELF-EMF and that were fed a diet with NAC supplementation for 1 week (Figure 5I) and was reduced in skeletal muscles from mice exposed to ELF-EMFs (0.1 or 1.0 mT) with a NAC-supplemented diet for 5 weeks (Figure 5J). 

One week of exposure to ELF-EMFs, inducing oxidative stress, resulted in the increased oxidative damage of lipids and proteins of treatment. This damage was mitigated by NAC supplementation, pointing to the role of antioxidants against ELF-EMF-induced oxidative stress. Over longer exposure periods, the oxidative damage seemed to diminish, indicating a potential adaptive response of the skeletal muscles. These findings suggest that the early induced oxidative damage by ELF-EMF was partially recovered after 5 weeks of exposure, and the presence of antioxidants could moderate these effects.

## 3. Discussion

Our previous studies investigated the biological effects of ELF-EMFs on muscle cells, revealing that short-term exposure modulates oxidative stress and influences intracellular Ca^2+^ signaling pathways, which are crucial for muscle differentiation. Long-term exposure to ELF-EMFs enhances the myogenic process by increasing gap junction intercellular communication activity, suggesting potential therapeutic applications of ELF-EMFs in muscle repair and regeneration [18,27]. Furthermore, considering that ELF-EMFs promote tissue repair in organs like bone, cartilage, and skin and enhance functional recovery after stroke, with efficacy depending on specific frequencies [13,14,15,16,17], the present pilot study aimed to investigate the possible beneficial impact of ELF-EMFs on the skeletal muscle of sedentary adult mice. The experimental design involved three groups of mice: a sham group (control group) and two groups exposed to ELF-EMFs at intensities of 0.1 mT or 1.0 mT. These field intensities were chosen to explore a range of biological responses covering both the levels commonly encountered in daily environments and higher intensities typically used in experimental studies and clinical applications of ELF-EMFs [14,34,35]. Indeed, 0.1–0.2 mT is considered the secure limit of non-dangerous intensities of ELF-EMF exposure [36]. The therapeutic use of ELF-EMFs was reported in numerous studies for the treatment and rehabilitation of different musculoskeletal pathologies (such as acute and chronic pain, fractures, osteoarthritis, and osteoporosis) using different stimuli, from ELF-EMFs to pulsed-radiofrequency electromagnetic fields, and exposure times [14,35,37]. In this study, the exposures were administered for 1 h per day, 5 days a week, over periods of either 1 week or 5 weeks, simulating protocols commonly used in magnetotherapy during rehabilitation protocols [14,37]. This approach allowed the assessment of both short-term and long-term effects of ELF-EMFs on skeletal muscle physiology. Importantly, the mice were fed a diet with or without antioxidant supplementation, using N-acetylcysteine (NAC). This aspect of the study aimed to investigate whether antioxidant supplementation could modulate the oxidative status and the changes induced by ELF-EMF exposure, given that reactive oxygen species (ROS) exerts multiple effects on cellular processes, including proliferation, apoptosis, and differentiation [22,23,24,25]. 

In all tested conditions, the first parameters monitored in vivo were the mice’s body weight and muscle strength, both related to skeletal muscle status considering that this tissue represents a very high percentage of the body mass. Consistent with previous studies [38,39], our findings indicated no significant changes in body weight in all tested samples. The lack of significant changes in body weight suggests that ELF-EMF exposure did not induce systemic metabolic alterations that could affect body mass. However, a transient significant change in muscle strength was observed in mice exposed to a 1.0 mT ELF-EMF. This transient variation could be linked only to an initial boost in muscle fiber recruitment and activation, which might not yet translate to long-term changes in an enhanced muscle mass or overall body weight.

Skeletal muscle homeostasis and regeneration are regulated by a complex network of signaling pathways, involving the key role of satellite cells. In response to different stimuli (electrical, mechanical, etc.), these quiescent cells rapidly become mitotically active and proliferate generating myoblasts, then differentiate and fuse to regenerate the new muscle fibers [40,41,42]. These processes are regulated by a sequential expression pattern of different transcription factors such as Pax7, which is essential for satellite cell activation, MyoD and myogenin for myogenic commitment, and finally, myosin heavy chain (MyH) and α-actinin, which are specific proteins for muscle differentiation. Our data showed that ELF-EMF exposure increased the expression of one of the key markers of satellite cell activation, suggesting a role of ELF-EMFs in modulating muscle remodeling and in enhancing satellite cells’ proliferative capacity, which is crucial for muscle repair and regeneration. The increase in Pax7 expression levels after ELF-EMF exposure was not dependent on NAC supplementation, and the NAC itself induced this increase. These effects were persistent up to the 5-week experimental period. This result is in accordance with other authors who observed the stimulation of cell proliferation triggered by ELF-EMF exposure in different cellular models [43,44]. In addition, the modulation of MyH expression levels, a terminal marker of muscle differentiation, indicated that ELF-EMFs and/or NAC may influence different stages of muscle differentiation. L’Honoré et al. [45] demonstrated that NAC increased the proliferative capacity of satellite cells in primary cultures while inhibiting differentiation. Also, in our experimental model, NAC promoted satellite cells’ activation and inhibited final muscle differentiation, supported by the significant reduction in MyH in the sham group and in the 0.1 mT ELF-EMF group after 1 week of treatment. Conversely, after 5 weeks of treatment, ELF-EMF exposure showed a pro-differentiation effect, inducing an increase in MyH expression levels in mice muscle samples that was not related to NAC supplementation, even if NAC alone appeared to simulate the effects of ELF-EMFs. However, no additive effect was observed with ELF-EMF exposure in muscles from mice that were fed with a NAC-enriched diet. The different response of NAC underscores the complexity of antioxidant interactions with muscle regeneration pathways and highlights the importance of temporal factors in therapeutic strategies involving antioxidants. However, the lack of an additive effect suggests that both stimuli (ELF-EMFs and NAC) are likely influencing the same pathways or mechanisms. NAC could be stronger, as it was continually present in the diet, and fully activate the pathway on its own, or the pathway may have a maximum response limit that is reached with NAC and leaves no space for an additional effect from ELF-EMF exposure. Further investigation into the specific pathways and their regulatory mechanisms would be necessary to fully understand the interactions between these stimuli.

Although, at the cellular level, ROS have been proposed to mediate the effects of ELF-EMFs [18,46,47], several studies have reported controversial results in in vivo investigations [2,48]. For instance, Manikonda et al. reported an increase in ROS levels and lipid peroxidation, coupled with a reduction in antioxidant defenses in various brain regions of young male Wistar rats after 90 days of continuous exposure to 0.05–0.1 mT ELF-EMFs, leading to aberrant neuronal functions [49]. Conversely, another study found that ELF-EMF exposure led to an increase in antioxidant enzyme activity in the blood plasma of Wistar rats. In this study, the authors hypothesized that ELF-EMF exposure induced low levels of ROS probably responsible for the protective effect due to cellular redox modifications [50]. 

However, it is now widely established by the scientific literature that ROS play a dual role in the physiology of skeletal muscle. At low concentrations, ROS can modulate cellular proliferation, differentiation, and muscle contraction, thus increasing muscle force and enhance adaptation to exercise, whereas at a high concentration, ROS lead to a decline in muscle performance [51,52]. Since ROS levels depend on the dynamic balance between ROS generation and removal, this study evaluated whether ELF-EMF exposure affected ROS production or removal processes. 

Our data show no significant changes in NOX2 and NOX4 expression or activity, which are considered one of the major sources of ROS in striated muscles [53]. However, it is noteworthy that there was an increase, although not significant, in NOX2 expression, particularly after exposure to a 0.1 mT ELF-EMF, observed as early as 1 of week treatment and persistent up to 5 weeks. NOX2 is predominantly located in the sarcolemma and transverse tubules, while NOX4 is also expressed in the sarcoplasmic reticulum and mitochondria [54,55]. NOX4 is constitutively active, contributing to the baseline production of ROS in myocytes. In contrast, NOX2 activation is induced by specific stimuli, such as mechanical or contractile stress. Moreover, a growing body of evidence suggests that NOX2 is directly involved in mechano-transduction in response to mechanical stimuli [53]. Therefore, we speculate that the observed slight increase in NOX2 expression could represent a sensible target to the presence of physical agents such as ELF-EMF, leading to an increase in ROS production. 

To further understand the implications of these findings, we evaluated the activation of antioxidant enzymes, which is indicative of ROS balance. This analysis is crucial to determine whether the observed trends in NOX2 expression correlate with compensatory mechanisms aimed at mitigating oxidative stress. The redox status of skeletal muscle was evaluated by analyzing the protein expression of antioxidant enzymes (SOD1, SOD2, catalase), the total antioxidant capacity (that includes enzymatic and non-enzymatic antioxidative factors), and the transcription factor NRF2, which regulates the expression of genes involved in antioxidant response.

Interestingly, after 1 week of treatment, an increase in the total antioxidant capacity of skeletal muscle from mice exposed to ELF-EMFs was observed that was not related to the presence of NAC in the diet and was accompanied by an increase in catalase expression levels and a decrease of its activity. These last effects were reduced by the presence of NAC in the diet. 

Taken together these results highlight an involvement of the oxidative metabolism in the ELF-EMF-induced effects and a possible adaptive mechanism to manage increased ROS production in response to ELF-EMF exposure. Indeed, the downregulation of catalase activity despite its increased expression could indicate a potential post-translational modification required as a homeostatic response. The presence of NAC seemed to counteract these effects, indicating its protective role against ELF-EMF-induced oxidative stress. NAC’s role as an antioxidant was also underscored by its ability to reduce elevated levels of lipid oxidation in response to 1 week of exposure to a 1 mT ELF-EMF, the only condition in which it is possible to observe oxidative-induced damage to lipids.

After 5 weeks of exposure, there were no significant differences in the antioxidant parameters even if a trend of increase in the total antioxidant capacity persisted. The results after 5 weeks of treatment indicated a potential adaptation to prolonged ELF-EMF exposure. This suggests that the skeletal muscle could achieve a new homeostatic balance following prolonged exposure. 

Remarkably, among the markers of oxidative-induced damage to macromolecules, the protein carbonyls appeared significantly increased in samples exposed to ELF-EMFs starting from 1 week of exposure, and NAC supplementation induced only a partial reduction of this effect, indicating that NAC can only mitigate oxidative damage induced by ELF-EMFs. One possible explanation for this could be the different mechanisms through which NAC interacts with lipid and protein oxidation processes. NAC is known to replenish intracellular levels of reduced glutathione, an essential non-enzymatic antioxidant that plays a significant role in maintaining cellular redox balance [56]. Studies on animal models have consistently shown that this antioxidant is associated with improvements in fatigue resistance [57,58] and has a beneficial effect on muscle regeneration and function [59]. However, it is important to highlight that several groups have found that NAC can provide protective benefits to skeletal muscle without significantly affecting biomarkers of protein and lipid oxidative damage [58]. In mdx mice, NAC administration successfully mitigated exercise-induced myonecrosis but did not prevent alterations in the levels of oxidative modifications to lipids and proteins within skeletal muscle [60]. These observations emphasize the notion that NAC may exert multifaceted positive effects on skeletal muscle, implying the involvement of alternative mechanisms beyond direct modulation of oxidative status in macromolecules.

## 4. Materials and Methods

### 4.1. Exposure System

ELF-EMFs (50 Hz and 0.1 or 1.0 mT) were generated by a solenoid (length: 300 mm and diameter: 124 mm; Oersted Technology Corp., Troutdale, OR, USA). The coil was made of copper (Ø: 1.25 mm), and the wire was made of a single layer, precisely winded to preserve field homogeneity. The coil carrier (length: 350 mm) was non-magnetic, with maximal thermal and mechanical stability, suitable for long-term operations (up to many days in a continuous operating mode). The power supply (Elgar Electronics, San Diego, CA, USA) was connected to the solenoid through a highly reliable cable and set following manufacturer’s instructions and checked by an appropriate probe [61].

### 4.2. Animals and Experimental Plan

This study was conducted on male C57BL/6 mice at the age of 12 weeks. The care and use of the mice strictly followed “The Guiding Principles for the Care and Use of Animals”, in accordance with the principles of the Declaration of Helsinki, with the European Community Council (86/609/CEE) and the Italian Government law on the protection of animals for experimental procedures in research laboratory (92/116). The mice were housed in the animal facility of the Center of Advanced Studies and Technologies (CAST) at the University G.d’Annunzio of Chieti-Pescara (Chieti, Italy).

All procedures were also approved by the local University Committee on Animal Resources, Comitato Etico Interateneo per la Sperimentazione Animale—CEISA (2011, prot. n. 03/2011/CEISA/PROG/11). 

Mice were randomly divided into three groups (n = 24/group, Table 1): one sham group and two ELF-EMF-exposed groups. 

**Figure 6:**
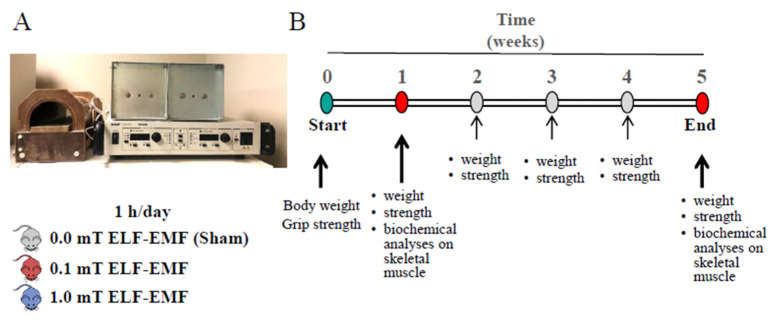
The setup and the experimental plan configuration. (**A**) Experimental set-up: the horizontal solenoid with power supply. The mice, one at a time, were placed inside the solenoid for 1 h/day for 5 days/week up to 1 week or 5 weeks. The solenoid worked at 0.0 mT for sham controls at 0.1 or 1.0 mT for mice exposed to ELF-EMFs. (**B**) Timeline of the experimental plan. During the experimentation, the mice were fed with a diet with or without NAC, and their weight and strength were recorded weekly. After 1 or 5 weeks, biochemical analyses were also performed.

All animals, one at a time, were placed inside the solenoid for 1 h/day for 5 days/week up to 1 week or 5 weeks, and the solenoid was turned off during sham control exposure and operated at 0.1 or 1.0 mT during ELF-EMF exposure for the exposed groups (Figure 6). 

Body weight and muscle strength of each mouse were monitored weekly over a period of 5 weeks (Figure 6B). At the end of 1 week or 5 weeks, the mice (n = 36 for 1 week and 36 for 5 weeks) were sacrificed by cervical dislocation, and skeletal muscles were collected from the hind limbs of each mouse. Then, muscle tissues of each mouse were rapidly frozen in liquid nitrogen and stored at −80 °C for subsequent biochemical analyses (proteins’ expression levels and enzymatic activities) were performed on extracts from skeletal muscles isolated from hind limbs. During the experimentation, both exposed and sham mice were fed a diet supplemented with or without N-acetylcysteine (NAC, Merck Life Science S.r.l., Milan, Italy). NAC was administered through ad libitum access to drinking water containing 1% weight/volume (1% *w*/*v*) NAC following the protocol described by Michelucci et al. [62]. At the end of the experimentation period (1 week or 5 weeks), after body weight and grip test measurements, sham and ELF-EMF-exposed mice were sacrificed.

### 4.3. Body Weight and Grip Strength Test

Body weight and grip strength measurements were performed as previously described by Caprara et al. [63]. Briefly, the mice were weighed before the grip strength test. Grip strength was evaluated using a force transducer (ShimpoFgv0.5×; Metrotec Group, San Sebastian, Spain) by lifting the mouse and allowing it to grasp a grid with its paws. Then, the mouse was gently pulled by the tail until it let go of the grid. The force of resistance was measured in a single three-trial session (with appropriate intervals, at least 30 s, to avoid fatigue), and the strongest measure was recorded as the best run. Peak force values were normalized to total body mass measured before grip strength test. 

### 4.4. Chemicals and Materials

Unless otherwise indicated, cell culture media, sera, antibiotics, and cell culture dishware were obtained from Thermo Fisher Scientific (Monza, Italy); reagents and standards were from Merck Life Science (Milan, Italy).

### 4.5. Western Blotting Analysis

Proteins were extracted from hind limb muscles of the mice using a protocol previously described by Caprara et al. [63]. Briefly, the tissues were homogenized (T10 basic Ultra Turrax IKA-Werke GmbH and Co. KG, Staufen, Germany) in ice-cold lysis buffer containing 50 mM Tris-HCl, 100 mM NaCl, 50 mM NaF, 40 mM b-glycerophosphate, 5 mM EDTA, 1% TritonX-100, 200 μM sodiumorthovanadate, 100 μg/mL phenylmethylsulfonylfluoride, 10 μg/mL leupeptin, 5 μg/mL pepstatin-A, 10 μg/mL benzamidine, pH 7.4. After centrifugation (10,000× *g*, 10 min, at 4 °C), the protein concentrations in the supernatants were quantified by Bio-Rad protein assay (Bio-Rad Laboratories S.r.l, Segrate (MI), Italy). Samples (40 μg) were resuspended in Laemmli buffer and separated by SDS-PAGE homogeneous slab gels and then electroblotted onto nitrocellulose membranes (Amersham™-Hybond™-ECL; GEHealthcare, Milan, Italy). After blotting, the membranes were blocked with EveryBlot Blocking Buffer (Bio-rad) for 15 min at room temperature before incubation with primary antibodies overnight at 4 °C. After washing, the membranes were incubated with the appropriate horseradish peroxidase-conjugated secondary antibodies (1:10,000 in blocking buffer) for 1 h at room temperature. The signals were detected using an ECL kit (AmershamTM Cytiva, Marlborough, MA, USA) and analyzed with an image acquisition system (Uvitec mod Alliance 9.7, Uvitec, Cambridge, UK). Primary antibodies used in this study are reported in Table 2.

### 4.6. Total Antioxidant Status

Total antioxidant capacity in skeletal muscle was measured by a colorimetric assay using a commercial kit (TEAC assay, Cayman Chemical, Ann Arbor, MI, USA) and following the manufacturer’s instructions. This assay is a widely used kit-based commercial method based on suppression of the absorbance of radical cations of 2,20-azino-di-(3-ethylbenzthiazoline sulphonate) (ABTS) by all antioxidants (including proteins, lipides, vitamins, glutathione, etc.) present in the test sample. The antioxidants’ capacity is compared to that of Trolox. In our study, total hind limb muscles were homogenized in cold buffer (5 mM potassium phosphate, pH 7.4, 0.9% NaCl, 0.1% glucose) and centrifuged (10,000× *g*, 15 min at 4 °C). Cytosolic proteins (50 µg), determined by Bio-Rad protein assay (Bio-Rad Laboratories), were processed following the manufacturer’s instructions. Reaction mixtures were read at 750 nm using a microplate reader (Synergy H1 multimode, Biotek, Bad Friedrichshall, Germany). Results were expressed as Trolox equivalents by reference to a linear calibration curve computed from pure Trolox-containing reactions (range 0–0.33 mM).

### 4.7. Catalase Assay 

The catalase activity was assessed with a spectrophotometric method using commercial kit (Sigma-Aldrich, St. Louis, MO, USA, Merck Life Science S.r.l.). Briefly, hind-limb muscles were homogenized in 50 mM potassium phosphate buffer, pH 7.0, containing 0.1% Triton X-100 and protease inhibitors (Thermo Fisher Scientific). The homogenates were centrifuged at 10,000× *g* for 10 min at 4 °C, and supernatants were collected for the enzymatic assay performed following the manufacturer’s instructions. The catalase present in the sample reacts with hydrogen peroxide (H_2_O_2_) to produce water and oxygen. The unconverted H_2_O_2_ reacts with probe to produce a product that can be measured colorimetrically at 570 nm. The catalase assay was initiated by mixing 5 μL of supernatant with 50 mM H_2_O_2_ in a total volume of 25 μL (reaction mix). The reaction was carried out at room temperature for 5 min and stopped with 15 mm sodium azide. The residual H_2_O_2_ was measured colorimetrically after incubation with Color Reagent for 15 min at room temperature. The reaction produced a red quinoneimine dye that absorbs at 520 nm. The concentration of H_2_O_2_ in the samples was determined using a standard curve. Results were expressed as the ratio between the concentration of remaining H_2_O_2_ and protein content of every sample.

### 4.8. NOXs Activity

The NOXs activity was evaluated through a colorimetric assay using a commercial kit (Abcam, Cambridge, UK). Total hind limb muscles were homogenized in sodium phosphate pH 7.0, containing protease inhibitors, and then centrifuged at 10,000× *g* for 10 min at 4 °C. Supernatants were collected and processed following the manufacturer’s instructions. The assay started by mixing each sample (50 μg) with NADH OXIDASE Enzyme mix, Substrate I, Substrate II, and Probe in a total volume of 100 μL. The assay was performed recording the absorbance at 600 nm in kinetic mode for 30 min at 25 °C using a microplate reader (Synergy H1 multimode, Biotek, Bad Friedrichshall, Germany). The oxidation of NADH by NOX and the reduction of a colored substrate leading to a colorless product was determined using a standard curve**.**

### 4.9. Protein Carbonyl Content

The total protein carbonyls, biomarkers of protein oxidation, were measured using a commercial kit based on a colorimetric assay (Protein Carbonyl Assay Kit, cat. 10005020, Cayman Chemical), following the manufacturer’s instructions and as previously described [18]. Briefly, the protein carbonyl content was assayed by incubating tissue extracts (200 μg, obtained as described above in Section 4.8) with 2,4-dinitrophenylhydrazine (DNPH), which reacted with protein carbonyls, forming a Schiff base to produce the corresponding hydrazone, which was analyzed spectrophotometrically at 370 nm using a molar adsorption coefficient for DNPH of 22,000 M^−1^ cm^−1^. Results were expressed as nanomoles of DNPH per micrograms of protein.

### 4.10. Statistical Analyses

The data, if not otherwise reported, are expressed as Means ± standard error of the mean (S.E.M.) of six independent experiments, each of which regarded the exposure of a mouse at 0.0, 0.1, or 1.0 mT for time periods and conditions described in the paragraph “Animals and experimental plan”. Statistical analyses were performed using Student’s *t*-test with Prism5 software (GraphPad, San Diego, CA, USA). *p*-values < 0.05 were considered statistically significant.

## 5. Conclusions

In conclusion, this study aligns with our previous findings in vitro [18,27], demonstrating that, in adult sedentary male mice, ELF-EMF exposure at mT intensities modulated the myogenic process, influencing the redox status of skeletal muscle, even if a dose-dependent effect was not evident. In this pilot study, we have shown that ELF-EMF exposure induced significant changes in skeletal muscle features. These were characterized by a transiently increased muscle strength, an initial upregulation of Pax7 activating satellite cells and of antioxidant enzymes, followed by possible adaptation mechanisms with prolonged exposure. Notably, the presence of NAC had different behaviors: some tested parameters appeared to mimic the ELF-EMF-induced effect, while others counteracted it. This underlies the different mechanisms activated by this antioxidant in vivo. 

Collectively, these findings suggest that ELF-EMF acts as a moderate cellular stressor that could trigger the production of ROS (potentially mediated by NOX enzymes), which serve as redox signaling messengers. This process involves both antioxidant mechanisms aimed at reducing oxidative damage and molecular pathways aimed at preserving and enhancing tissue homeostasis through muscle regeneration.

The ability of ELF-EMFs to stimulate antioxidant pathways further underscores their potential therapeutic value. ELF-EMFs could help limit the increase (or high levels) in ROS implicated in many muscle pathologies, from age-related sarcopenia to muscle degeneration seen in chronic diseases. Additionally, the effects of NAC observed in this study open up possibilities for combined therapies where ELF-EMFs and specific antioxidants could be tailored to enhance therapeutic outcomes, especially in conditions characterized by oxidative stress or impaired muscle regeneration.

The results presented here represent a preliminary snapshot of the behavior of skeletal muscle after in vivo ELF-EMF exposure, considering the multifaced aspect. Future research should aim to elucidate the detailed molecular pathways responsible for these effects and explore the therapeutic potential of ELF-EMFs and antioxidants in promoting muscle health and regeneration. 

Sequels of this study should have different specific focuses concerning not only the choice of the ELF-EMF intensities and times of exposure but also the administration of potential enhancing or counteracting factors (such as antioxidants or physical training). These studies could be developed in different physiological (i.e., aging, youth, exercise) or pathological (i.e., acute and chronic pain, post-traumatic atrophy, osteoporosis, etc.) conditions. 

## 6. Limitations and Future Directions

The experimental plan of this study represents a pilot investigation with a small-scale set of observations.

One of the primary restrictions of this study is the limited sample size, aimed only at having a rapid screenshot of a possible inference of ELF-EMFs on skeletal muscle using in vivo exposure conditions. This could not fully capture the short and long-term effects and the variability of responses to ELF-EMFs. Additionally, we assessed the impact of ELF-EMFs on muscle differentiation/regeneration processes as well as on oxidative stress markers and antioxidant responses, but the detailed molecular mechanisms underlying these changes remain to be elucidated. Future studies should aim to investigate the signaling pathways involved in in vivo ELF-EMF-induced oxidative stress and the role of other cellular defense mechanisms.

Furthermore, our study focused on sedentary adult mice, which may not represent other physiological (such as aging or active lifestyles) or pathological conditions. Exploring the effects of ELF-EMFs in different physiological or pathological contexts and in conjunction with other environmental factors will provide a more comprehensive understanding of their biological impact and therapeutic role.

## Figures and Tables

**Table 1:** Experimental groups.

Group	Exposure Intensity	Exposure Time	Antioxidant Supplementation	Number of Mice
Sham n = 24	0.0 mT	1 week	w/o 1% NAC	6
			with 1% NAC	6
		5 weeks	w/o 1% NAC	6
			with 1% NAC	6
ELF-EMF n = 48	0.1 mT	1 week	w/o 1% NAC	6
			with 1% NAC	6
		5 weeks	w/o 1% NAC	6
			with 1% NAC	6
	1.0 mT	1 week	w/o 1% NAC	6
			with 1% NAC	6
		5 weeks	w/o 1% NAC	6
			with 1% NAC	6

**Table 2:** Primary antibodies used for Western blot analyses.

Antibody	Working Dilution	Company and Reagent Code
Mouse monoclonal antibody anti-PAX7	1:1000	Santa Cruz Biotechnology Inc., SantaCruz, CA, USA, cod. sc-81648
Mouse monoclonal antibody anti-MyoD	1:1000	Santa Cruz Biotechnology Inc., cod. sc-377460
Mouse monoclonal antibody anti-MyoG	1:1000	Santa Cruz Biotechnology Inc., cod. sc-12732
Mouse monoclonal antibody anti-α-actinin	1:1000	Merck Life Science S.r.l, cod A7732
Mouse monoclonal antibody anti-MYH	1:1000	Santa Cruz Biotechnology Inc., sc-376157
Rabbit polyclonal antibody anti-SOD1	1:1000	Thermo Fisher Scientific, cod. PA527240
Muse monoclonal antibody anti-SOD2	1:1000	Thermo Fisher Scientific, cod. MA5-31514
Rabbit monoclonal antibody anti-catalase	1:1000	Cell Signaling Technology, Pero, Italy, cod. 14097
Rabbit monoclonal antibody anti-NRF2	1:500	Merck Life Science S.r.l, cd SAB4501984
Rabbit polyclonal antibody anti-pNRF2	1:1000	Thermo Fisher Scientific, cod PA567520
Mouse monoclonal antibody anti-NOX2	1:500	Santa Cruz Biotechnology Inc., cod. sc-130543
Rabbit monoclonal antibody anti-NOX4	1:500	Thermo Fisher Scientific, cod. MA5-32090
Mouse monoclonal antibody anti-4HNE	1:1000	Merck Life Science S.r.l., cod. SAB5202472
Rabbit polyclonal antibody anti-3-nitrotyrosine	1:1000	Merck Life Science S.r.l., cod. 4511
Mouse monoclonal antibody anti-β-tubulin (used as loading control)	1:1000	Thermo Fisher Scientific, cod MA5-16308
Mouse monoclonal antibody anti-GAPDH (used as loading control)	1:10,000	Merck Life Science S.r.l., cod. CB1001

## Data Availability

The data supporting the conclusions of this article will be made available by the authors on request.
